# Transgenic *Arabidopsis thaliana* and *Nicotiana tabacum* overexpressing the *Eucalyptus grandis* Cellulose Synthase 3 and its expression pattern in different *Eucalyptus *species and tissues

**DOI:** 10.1186/1753-6561-5-S7-P167

**Published:** 2011-09-13

**Authors:** Wesley L Marques, Marcela Salazar, Eduardo Camargo, Jorge Lepikson-Neto, Danieli Cristina Gonçalves, Leandro Costa do Nascimento, Carla Garcia, Adriano Almeida, Gonçalo Pereira

**Affiliations:** 1 State University of Campinas – UNICAMP, Brazil; 2 International Paper

## Background

In Brazil, the forest industry accounts for 4,5% from the Gross Domestic Product and the country is the biggest *Eucalyptus* cellulose exporter. That’s really good news because *Eucalyptus *forests are a competitive and efficient alternative to convert carbon from the atmosphere in cellulose, an important source for paper and bioenergy production.

The cellulose biosynthesis happens through the *Cellulose Synthase Complex* activity. This complex is composed by different *Cellulose Synthase* genes (*CesA*) that work together in a non redundant way [1]. It is also known that some of these isoforms act in the primary cell wall synthesis while others, in the secondary cell wall. In this last group there is the gene *Eucalyptus Cellulose Synthase 3* (*EgCesA3*), the most expressed *CesA* gene during xylogenesis [2]. Besides, knockout experiments proved that the *AtCesA7* (*EgCesA*3 ortholog in *Arabidopsis thaliana*) activity is essential for the xylem formation and for plant vertical growth [3].

In front of these evidences, the *EgCesA3* gene had it expression pattern evaluated in leaf and xylem tissues among the three most economic important *Eucalyptus* species in Brazil: *E. grandis*, *E. globulus* and*E. urophyla.*

As demonstrated in this work, the expression experiment provided enough information about the *EgCesA3* function, that’s why this gene was chosen to be overexpressed in model plants (*Arabidopsis thaliana* and *Nicotiana tabacum*). It’s expected increased cellulose content in the transgenic plants xylem.

## Material and methods

The *EgCesA3* expression pattern was examined trough qRT-PCR and Northern-Blot in xylem and leaf from the tree most commercially important *Eucalyptus* species: *E. grandis*,*E. urophyla* and*E. globulus*. The qRT-PCR was performed using SYBR Green and amplicons with approximated size of 100pb. On the other hand, the Northern-Blot was made with probes containing phosphorous-32 radioactive.

To create transgenic plants with improved cellulose content this work overexpressed the gene *EgCesA3* under the control of the CaMV 35S promoter in *A. thaliana* and *Nicotiana tobacum* trough *Agrobacterium* transformation by floral-spray.

## Results and discussion

**Figure 1:**
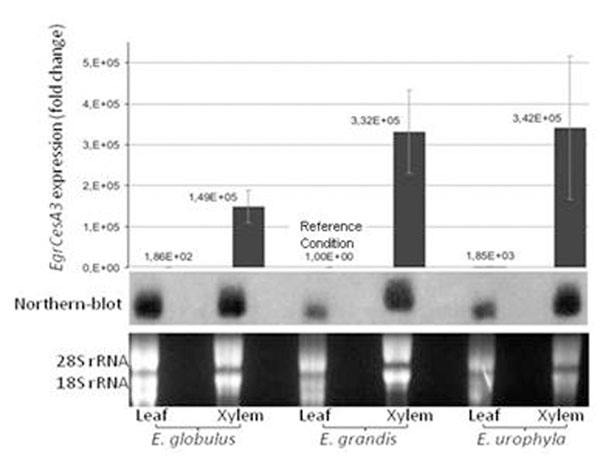
*Eucalyptus Cellulose Synthase 3* expression pattern. On the top, expression pattern analyzed trough qRT-PCR and, below, the expression analyzed trough Northern-Blot. The tissues studied were leaves and xylem from three different *Eucalyptus* species: E*. globulus*, *E. grandis* and *E. urophyla.*

The results show that the *EgCesA3* is strongly more expressed in xylem than in leaves among the three most important economical *Eucalyptus* species for the Brazilian forest industry (Fig. 1). This data corroborate to the theory that this gene is related to the secondary cell wall formation during the xylem development.

However, it’s necessary to mention that the gene expression data obtained through the northern-blot related to the *E. globulus* leaf can’t be analyzed. This because the amount of RNA used in this sample was different from the others. It can be seen through the rRNA amounts in the RNA electrophoresis (Fig. 1).

Besides, comparing the*EgCesA3* expression pattern between the xylem from the three species studied, it’s possible to conclude that should exist a difference: the *EgCesA3* gene is apparently more expressed in *E. grandis* and *E. urophyla* xylem than in *E. globulus* xylem (Fig. 1). However, this conclusion isn’t distant from doubts: the problem is the large error bars that happened in the experiment. In order to solve it, the qRT-PCR repetition is being carried on.

**Figure 2:**
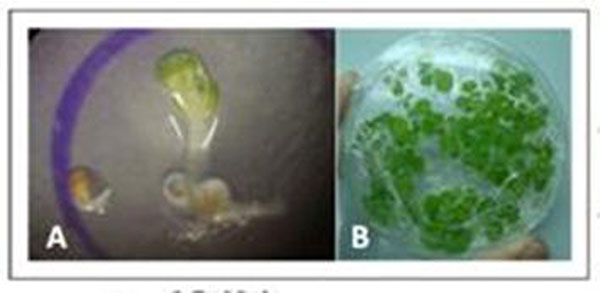
Transgenic model plants overexpressing the *Eucalyptus grandis Cellulose Synthase 3* gene. In “A” it’s possible to see one transgenic *A. thaliana* near a nother non-transgenic seeds being selected in the media containing hygromycin. In “B” it’s shown transgenic *Nicotiana tabacum* growing in selection media with hygromycin.

In front of the *EgCesA3* rolein the secondary cell wall synthesis, this gene was overexpressed in model plants (Fig. 2). The genetic modified plants were successfully obtained. Thus, after the generation advance, the homozygous transgenic plants will be evaluated to measure the cell wall chemical composition and morphology.

## Conclusion

The *Eucalyptus Cellulose Synthase 3* function in the secondary cell wall synthesis was confirmed by this work;

The cellulose content variation trough the species studied should be a consequence of the *Eucalyptus CesA3* expression level;

Transgenic *A. thaliana* and*N. tabacum* overexpressing the *EgCesA3* gene were successfully obtained. The transgenic plants analyses are being performed; however it’s already possible to notice, by empirical view, growth improvement in the transgenic plants in comparison to the wild type.
